# Supplementary material for: Are carbon emissions trading and green financial instruments synergistic? -Comprehensive quantitative research based on content analysis
Source: PLoS One. 2024 Mar 7;19(3):e0298601. doi: 10.1371/journal.pone.0298601 (PMC10919601; doi:10.1371/journal.pone.0298601)
Supplement: S1 Data — (ZIP) [file pone.0298601.s001.zip › Data/地级市Z_F工作报告.zip!/╘╞─╧/┴┘▓╫╩╨/└┤╫╘╣1⁄2╓┌║┼í╛┬φ┐╦╩2╛▌═°í┐_2018.docx]

各位代表：

　　现在，我代表市人民政府，向大会报告工作，请予审议，并请政协委员和列席人员提出意见。

　　一、2017年及第三届市人民政府工作

　　2017年，在省委、省政府和市委的坚强领导下，在市人大及其常委会的法律监督、工作监督和市政协的民主监督下，市人民政府深入学习宣传贯彻党的十八大、十九大精神，以习近平新时代中国特色社会主义思想为指导，对标习近平总书记对云南发展的重要指示精神，认真贯彻落实陈豪书记、阮成发省长到临沧调研的指示要求，统筹推进“五位一体”总体布局和协调推进“四个全面”战略布局，落实新发展理念，立足临沧发展的三个阶段性特征，坚持稳中求进工作总基调，全力稳增长、促改革、调结构、攻脱贫、惠民生、防风险、强稳定，全市经济稳中有进。实现地区生产总值604.1亿元，增长10%；固定资产投资1170亿元，增长27.6%；地方一般公共预算收入40亿元，增长5.7%；地方一般公共预算支出242.9亿元，增长13.9%；社会消费品零售总额195.3亿元，增长12.5%；外贸进出口总额52.7亿元，增长15.1%；城镇常住居民人均可支配收入25056元，增长8.6%；农村常住居民人均可支配收入9814元，增长10.1%；金融机构存款余额605亿元，增长4.9%，贷款余额485.4亿元，增长9.5%。圆满完成了目标任务。

　　（一）着力稳增长促跨越，产业发展取得明显成效。积极从中央和省重要会议的工作报告，以及中央、省领导的指示和产业政策中找项目，梳理项目1078个，开工建设708个。全力抓好投资工作，滚动实施“四个一百”重点项目。出台23条稳增长措施，投放国家专项建设基金32.9亿元。以供给侧结构性改革为主线的8大领域26项年度重大改革稳步推进，“三去一降一补”重点工作有效落实。粮食总产量达106.8万吨，实现十连增。新增农业产业化基地36万亩。临沧普洱茶产区被列为中国特色农产品优势区，双江勐库大叶种茶、镇康马鞍山茶、博尚菜籽油获得国家地理标志登记（商标）保护。不动产统一登记制度改革顺利推进，农村集体土地确权、集体建设用地和农村宅基地确权颁证工作基本完成。打响工业经济攻坚战。抓好企业帮扶解困，为企业提供1.2亿元应急互助资金，支持企业银行调头资金1.9亿元。新增规模以上企业18户，规模以上工业增加值达87.4亿元，增长13.1%。扶持微型企业1100户，民营经济增加值增长10.6%。现代服务业加快发展。电子商务进农村综合示范项目覆盖8县（区）。成功举办第十三届中国茶业经济年会暨“天下茶尊·红茶之都”云南临沧红茶节、临沧美食大赛、第五届亚微节暨第七届边交会、第五届云南航空旅游市场推介会，旅游业总收入177.4亿元，增长58.3%。

　　（二）着力改善基础设施，“五网”建设实现新突破。大理至临沧铁路完成投资11.6亿元，临沧至清水河铁路完成工可审查，临沧至普洱、临沧至芒市铁路和临沧城市轨道交通前期工作加快推进。临沧机场高速公路建成通车，结束了临沧无高速公路的历史。玉溪至临沧、临翔至清水河、临翔至双江、南伞至清水河高速公路完成投资30.3亿元。云县至临翔、云县至凤庆高速公路控制性工程加快推进，链子桥至勐简高速公路开工建设。小黑江至勐省二级公路建成通车。启动直过民族、沿边自然村道路硬化工程。完成建制村通畅工程695公里。凤庆机场、永德机场加快建设。水利项目数量、投资规模为历年之最，水利水电固定资产投资突破100亿元。对缅境外输电网建设加快推进，中缅天然气管道（临沧）支线项目签订特许经营协议，云县光伏电站、凤庆县农光互补项目投入运行。互联网等通信网络不断完善。

　　（三）着力完善财税金融服务体系，发展支撑显著增强。成功搭建宏观金融合作关系支撑的融资平台，与42家金融机构成功建立金融战略合作关系，达成未来5年可对接协议资金3000亿元，累计投放资金达350亿元，其中通过市金融办实现的进账资金120亿元。加快设立母基金和各类专项基金，互利共赢的良性投融资体系初步建立。在市国资公司建立的财务结算中心（资金池）开始运行，实现第一阶段的资金归集目标。一批新的金融工具引入临沧。富滇银行临沧分行开业运营，农村信用社改制农商行稳步推进。全市基本形成了8家银行机构、16家保险机构、1家证券机构和其他金融机构互为支持的金融组织体系。加强金融风险防控工作，金融环境持续优化。财税体制改革不断深化。

　　（四）着力抓好脱贫攻坚，民族团结进步事业迈上新台阶。着力动态管理，年初有建档立卡贫困人口4.2万户、15万人。扎实开展“找问题、补短板、促攻坚”专项行动。农村危房改造、易地扶贫搬迁扎实推进，教育、卫生与健康、产业发展等精准扶贫政策有效落实。实施“健康扶贫”30条措施，努力做到群众“看得起病、方便看病、看得好病、尽量少病、病不致贫”。贫困人口基本医疗保险参保率100%、养老保险参保率100%。深入实施人口较少民族、直过民族脱贫攻坚计划，第二轮“十百千万示范创建工程”三年行动计划，民族团结进步示范市创建工作顺利推进。云县、耿马、双江3个贫困县脱贫摘帽通过市级初审，并上报省级复核，10个贫困乡镇、161个贫困村、9.5万贫困人口如期脱贫。目前，有建档立卡贫困人口2.5万户、8.6万人。

　　（五）着力推进“四个先行”，解决“五通”问题取得新进展。认真落实“一带一路”国际合作高峰论坛成果，梳理出120个项目，启动建设76个。孟定口岸新城、综合交通枢纽示范城市规划建设加快推进，清水河口岸迁址新建项目顺利实施，国际贸易“单一窗口”推广工作有效开展，通关便利化不断提高。缅甸滚弄大桥援建项目获国务院批复、立项。与缅甸腊戍市签署友好城市意向书。3613名缅籍学生到我市各类学校就读。深化沿边金融综合改革，设立19个跨境金融服务点，数量居全省首位。招商引资市外到位资金1200.9亿元，增长18.9%。

　　（六）着力提升城乡人居环境，居住环境质量不断改善。创造性实施“河长、湖长、街长、路长、园长、山长”六长制，共设“六长”6530个，沿江沿河沿路沿街重点区域环境明显改善。以“四治三改一拆一增、七改三清”为重点，城乡脏乱差现象得到较大整治。打通主城区“断头路、丁字路”24条（段）。实施“增绿、增花、增果、增水、增光”工程，累计增花97万平方米，增果440万株，增水3万平方米，增光2.8万盏，城市绿地率达35%。临沧城市规划馆正式投入使用。提升完善了66个乡镇规划。改造旧住宅区41万平方米、旧厂区18万平方米、棚户区2.46万户。深入开展“拆违”“拆临”专项行动，查处违法建筑107万平方米。新开工棚户区改造住房4242套。分配入住公租房8742套。住房公积金制度覆盖范围不断扩大，普惠性金融保障功能进一步彰显。乡镇自来水覆盖率达89.7%，污水处理设施覆盖率达67.7%，生活垃圾处理设施覆盖率达98.5%。155个建制村实现污水治理，7097个自然村实现垃圾有效治理。提升抵边群众人居环境。双江勐库镇列入第二批全国特色小镇，6个特色小镇列入全省创建名单并开工建设。

　　（七）着力推进森林临沧建设，生态文明建设取得新进展。临沧获“国家森林城市”和首批“全国森林旅游示范市”称号，镇康县被评为国家园林县城。实施水土流失等生态综合治理工程，完成新一轮退耕还林还草31.2万亩，陡坡地生态治理2万亩。实行古树（茶树）、名树挂牌保护。深化集体林权制度改革，加快推进国有林场改革、自然保护区管理体制改革。争取国家可持续发展议程创新示范区工作顺利推进。中央和省级环保督察、国家土地例行督察发现问题整改完成阶段性目标。推进煤炭行业和非煤矿山转型升级。节能减排等指标均在控制范围内。全市空气质量全年保持优良。

　　（八）着力保障和改善民生，人民群众生活水平不断提高。全面推进幸福临沧建设，民生支出占地方一般公共预算支出的81.8%。中小学“全面改薄”工程全面实施，义务教育巩固率达93.5%。普通高中教育扩容提质，高考一本上线率居全省前列，学前教育、职业教育、特殊教育、高等教育、成人教育、民办教育加快发展，劳动力平均受教育年限达10.3年。就业创业形势总体良好，劳动者权益保障有效落实。社会救助水平不断提高。紧密型县乡村医疗卫生服务一体化模式加快推广。城乡居民基本医疗保险覆盖率达100%。临沧文化中心投入使用，现代公共文化服务体系建设取得新成效。争做新时代“红色文艺轻骑兵”、医疗扶贫基地等做法被中央电视台报道，广播电视覆盖率达99.2%。群众体育蓬勃开展。高度重视安全生产，妥善处置了“3·02”重大道路交通事故。防灾减灾工作有序推进，8县（区）建成地质灾害防治高标准“十有县”。食品药品安全监管扎实有效。全力以赴抓好中缅边境“3·06”事件处置工作，劝返缅籍边民5.7万人。“平安临沧”创建活动深入开展，各类矛盾纠纷有效化解，第四轮禁毒防艾人民战争深入推进，群众安全感、满意度明显提升，社会更加和谐稳定。人民武装、双拥工作和人民防空事业取得新成绩。10件惠民实事全面落实。

　　各位代表，第三届市人民政府在市委的坚强领导下，主动服务和融入国家发展战略，凝心聚力，攻坚克难，全面完成国民经济和社会发展“十二五”规划目标任务，启动实施“十三五”规划，经济平稳向好、社会和谐稳定、人民安居乐业，支撑临沧跨越发展的“四梁八柱”初步形成，实现了圆满收官，为全面建成小康社会、开启新征程奠定了坚实基础。

　　——我们坚定不移抓发展第一要务，经济发展质量效益不断提升。正确认识、妥善处理经济总量、规模、速度和结构、质量、效益的关系，以供给侧结构性改革为主线，全力打好县域经济、园区经济和民营经济“三大战役”，经济综合实力明显增强。地区生产总值年均增长10.4%。投资、消费、出口协同发力，固定资产投资年均增长29.4%，社会消费品零售总额年均增长12.8%，外贸进出口总额年均增长10.5%。坚定不移走“两型三化”产业发展路子，农业产业化基地达2187万亩，农业龙头企业达157户，农业增加值年均增长6.1%，工业增加值年均增长11.7%，第三产业增加值年均增长10.8%。三次产业结构由30.6:32.9:36.5调整为26.9:34.4:38.7。

　　——我们坚定不移打基础破瓶颈，以综合交通为主的“五网”基础设施建设取得重大进展。启动“五网”建设5年大会战，铁路、高速公路建设实现“零”的突破。大理至临沧铁路、玉溪至临沧高速公路、临翔至清水河高速公路相继开工建设，临沧至清水河铁路、临沧至普洱铁路、临沧至芒市铁路进入国家中长期铁路网规划。“县县通高”规划建设顺利启动。建制村通畅工程全面完成。沧源佤山机场建成通航，临沧机场改扩建进展顺利。澜沧江航道整治工程加快推进。重点水利工程新开工26件、竣工16件，建成山区“五小水利”工程7.4万件，有效灌溉保障率由42%提高到49.5%。改造中低产田地107.2万亩，划定基本农田621.7万亩。新增光伏发电装机7万千瓦。完成500千伏博尚变电站扩建工程。城乡用电同网同价。宽带和4G网络实现建制村全覆盖。

　　——我们坚定不移推进美丽家园建设，城乡人居环境明显改善。优化国土空间开发格局，成为全省首个国家可持续发展实验区。牢固树立绿水青山就是金山银山的理念，贯彻落实《临沧市古茶树保护条例》《临沧市南汀河保护管理条例》，加强生态环境保护与治理，全市森林覆盖率达64.7%，城市垃圾处理率达95%，污水处理率达90%，县城和重点乡镇集中式饮用水源地水质优良率达100%。深入实施“一轴两城三带”“1+8”城镇发展战略、乡镇规划建设管理三年行动计划。建设保障性住房7.9万套，城镇建成区面积增加15平方公里，城镇化率提高6.1个百分点，达到40%。全力推进“洁净临沧”“美丽家园”“美丽乡村”建设，启动国家卫生城市创建工作，完成旧村改造3071个，危房改造22.4万户，美丽村庄提升800个，45.4万户农户住上了安居房。

　　——我们坚定不移推进对外开放，对缅合作开创新局面。坚定不移用习近平总书记“一带一路”、辐射中心战略思想统领对外开放，以“四个先行”推动对缅开放，争当对缅开放排头兵。边合区、孟定口岸纳入国务院沿边重点地区支持名录。清水河至缅甸登尼二级公路建成通车，中缅边境3条国际旅游线路开通运营。清水河口岸获批国家进口粮食指定口岸、全省第一个持护照通行的对缅陆路口岸。沿边金融综合改革试验区建设取得新业绩。33户企业投资缅北，建成产业基地116.8万亩。成立驻缅甸腊戍、内比都商务代表处。积极参与南博会，成功举办边交会、亚微节等活动。招商引资市外到位资金年均增长23.9%，累计利用外资3698亿元。

　　——我们坚定不移保障和改善民生，社会事业全面进步。全面实施“27241”脱贫攻坚工程，深入实施兴边富民工程改善沿边群众生产生活条件三年行动计划，24.9万贫困人口实现脱贫。义务教育均衡发展，边境县14年免费教育取得突破。滇西科技师范学院挂牌成立。卫生与健康事业不断进步，“全面两孩”政策有序实施，社会保险提标扩面。社会救助体系不断完善。城镇登记失业率均控制在4%以内，居民收入稳步增长。现代公共文化服务体系加快构建，各类文体赛事步入高峰期。民族团结进步示范区建设取得新成绩。“法治临沧”“平安临沧”建设不断深入，禁毒防艾工作取得新成效，信访工作进一步加强。安全生产形势持续好转，食品药品安全监管不断加强。顺利完成震后恢复重建工作。军民融合深度发展，连续三届荣获“全国双拥模范城”称号。充分运用党政军警民“五位一体”协调联动机制，妥善做好中缅边境“2·09”事件处置及后续工作，有力维护了国家利益、边疆稳定、边境安宁。统计、调查、新闻、科技、保密、地方志、档案、供销、外事、侨务、对台、消防、海关、检验检疫、测绘、地震、气象、水文、参事、文史、哲学社会科学等工作不断加强，青年、妇女、儿童、老年人、残疾人、红十字会等事业健康发展。

　　——我们坚定不移加强政府自身建设，服务型政府建设成效显著。坚持党对政府工作的领导，深入开展“党的光辉照边疆，边疆人民心向党”实践活动，坚决提高政治站位，牢固树立“四个意识”，坚决维护习近平总书记在党中央和全党的核心地位，维护党中央权威和集中统一领导。严格落实主体责任，自觉接受各界监督，坚持正风肃纪，三公经费逐年下降，廉洁政府建设成效显著。重大项目督导巡察机制不断完善。及时办结人大代表建议和政协提案，被第十一届省政协表彰为提案工作先进单位。法治政府建设取得积极进展，严格决策程序，强化决策合法性审查。完成新一轮政府机构改革。规范行政执法权，“双随机一公开”监管方式全面推开。深化“放管服”改革，政务服务网上大厅上线运行，公共资源实现电子化交易，全面推行政府权力清单和责任清单制度，市级行政审批事项缩减282项。

　　各位代表，2014年以来，在市委的坚强领导下，全市各族干部群众砥砺奋进，赶超跨越，取得了许多历史性、开创性的重大成就，解决了许多难题，办成了许多大事。这些成绩，是习近平新时代中国特色社会主义思想引领的结果，是党中央、国务院，省委、省政府和市委坚强领导的结果，是市人大及其常委会的法律监督、工作监督和市政协民主监督的结果，是全市各族人民团结奋斗的结果。在此，我谨代表市人民政府，向全市各族人民，向人大代表、政协委员、各民主党派、工商联和无党派人士、各人民团体、离退休同志、各界人士，向驻临部队官兵、中央和省驻临单位，向所有关心和支持临沧发展的海内外友好人士，表示崇高敬意和衷心感谢！

　　各位代表，在充分肯定成绩的同时，我们也清醒地认识到，临沧加快发展面临着诸多困难和问题：基础设施滞后，大项目少、融资难；工业基础不牢，农业缺乏龙头带动，产业集聚效应不明显；沿边开放的层次和水平不高，实现“五通”任重道远；财政收入结构不合理，收支矛盾突出；重点工作压力较大；部分干部服务能力、履职能力有待提高，工作作风还需进一步转变。这些问题，需要在下步工作中认真研究解决。

　　二、今后五年的主要任务

　　今后五年，是全面脱贫、全面建成小康社会的决胜期，也是“两个一百年”奋斗目标的交汇期，临沧将迎来千载难逢的发展机遇：党的十九大精神、习近平新时代中国特色社会主义思想、习近平总书记对云南发展的重要指示，为我们推进跨越发展提供了行动指南，随着国家“一带一路”、辐射中心建设的深入推进，以及重大政策的落实，我市无可替代的优势进一步彰显，我们将获得国家、省更多的支持和帮助。同时，有历届市委、市政府团结和带领全市各族人民打下的坚实基础。我们必将把握好机遇，奋力赶超，实现跨越式发展。

　　今后五年的总体思路是：以习近平新时代中国特色社会主义思想为指导，围绕党的十九大确定的战略目标，深入落实习近平总书记对云南发展的重要指示精神，认真落实省委、省政府和市委决策部署，坚持稳中求进工作总基调，坚持新发展理念，按照高质量发展的要求，统筹推进“五位一体”总体布局和协调推进“四个全面”战略布局，坚定贯彻科教兴国战略、人才强国战略、创新驱动发展战略、乡村振兴战略、区域协调发展战略、可持续发展战略、军民融合发展战略，推动新型工业化、信息化、城镇化、农业现代化同步发展，打好防范化解重大风险、精准脱贫、污染防治攻坚战，努力实现地区生产总值年均增长10%以上，全面建成小康社会，谱写临沧跨越发展新篇章。

　　今后五年，我们将从九个方面全力推进临沧经济社会发展实现新跨越。

　　（一）坚定不移贯彻新发展理念，确保经济发展规模质量效益实现新跨越。着力投资，推进供给侧结构性改革，增加有效供给，实现固定资产投资年均增长20%以上。按照“两型三化”产业发展要求，打造“大产业”、培育“新主体”、建设“新平台”，综合开发优势资源，深入实施创新驱动和质量强市战略，推动互联网、大数据、人工智能和实体经济深度融合，打造“绿色能源”“绿色食品”“健康生活目的地”这三张牌，走出一条一二三产业融合发展新路子。每个县（区）聚力打造1—2个优势主导产业，做优做大“茶、果、糖、菜”等优势产业，巩固提升核桃、坚果、烤烟、咖啡、橡胶、畜牧等传统产业。争创国家农产品质量安全监管示范市，推进有机产品认证，打造一批知名的“临农”品牌。重点建设边境经济合作区、临沧工业园区、凤庆滇红生态园区、云县光伏园区、耿马绿色工业园区，拉动其他园区及水电工业、食品加工、生物制药、新技术锗业、风情美食、森林旅游等产业发展。抓实企业帮扶解困，培育新增一批规模以上工业企业。聚焦电子商务、康体休闲、养生养老等领域，大力发展加工贸易等现代服务业，加快对外贸易优化升级。抓住全省旅游产业转型升级契机，加快发展生态文化旅游业。

　　（二）强力推进交通强市战略，确保基础设施建设实现新跨越。基本建成互联互通、保障有力的路网、航空网、能源保障网、水网、互联网基础设施网络，把临沧建设成为国家战略的重要支撑节点。到2020年，基本实现“县县通高”目标。全面完成临沧机场改扩建工程，建成凤庆、永德机场。到2022年，建成临沧至清水河、临沧至普洱铁路，争取开工建设双江至澜沧、双江至沧源、巍山至凤庆至永德至镇康、云县至昔归高速公路。加快建设澜沧江航运通道。以中小型水库和水系、水源连通工程为支撑，构建水供给安全保障体系。发展光伏发电、风力发电。实施8县城和部分集镇天然气工程。加快宽带网络普及提速、三网融合发展，不断夯实“互联网+”基础，建设“智慧临沧”。

　　（三）全力打造辐射中心重要门户，确保沿边开放实现新跨越。坚持“四个先行”，促建经贸合作、科技创新项目孵化、金融服务、人文交流四个平台。争取中缅两国共建边境合作区，完成滚弄大桥援建项目，推动高速公路、铁路向境外延伸。加快沿边金融综合改革，推进孟定口岸新城、综合交通枢纽示范城市建设。充分发挥商务、外事、侨务等部门作用，深化对缅政策沟通和人文交流。着力构建跨境旅游互动机制、跨境金融服务互动机制、双方合作项目协调服务机制、通关便利化双方会商机制、双方互访常态化工作机制、招商激励机制，引进各类企业入驻边合区。到2022年，对缅互联互通基础设施建设、通关便利化及交流合作取得重大突破，临沧逐步成为辐射中心建设的主要通道、重要基地和支撑平台，基本形成对缅开放新格局。

　　（四）深入实施乡村振兴战略，确保城乡融合发展实现新跨越。坚持精准扶贫、精准脱贫，坚决打赢脱贫攻坚战，到2020年，8县（区）全部脱贫摘帽。坚持农业农村优先发展，按照“产业兴旺、生态宜居、乡风文明、治理有效、生活富裕”的总要求，编制乡村振兴战略规划，加快推进农业农村现代化。稳定农村土地承包关系，深化农村改革，壮大农村集体经济。推进农业供给侧结构性改革，构建高原特色现代农业产业体系，发展休闲观光型农业、园区复合型农业、洁净型农业，打造秀美乡村、美丽田园，建成一批现代农业产业园、田园综合体，促进农村一二三产业融合发展。改善乡村治理，传承优秀乡村文化，提高乡村德治水平。紧扣“特色、产业、生态、易达、宜居、智慧、成网”七大要素，推动县城和特色小镇发展，提升以人为核心的新型城镇化水平。到2022年，全市城市建成区面积增加20平方公里，20%以上建成区建成海绵城市，8县（区）创建为省级以上园林城市。深入实施城乡人居环境提升行动，落实“五长制”，继续实施城乡“四治三改一拆一增”、农村“七改三清”环境整治工程，不断提升城乡人居环境质量。

　　（五）强化信用建设和风险管控，确保金融支持保障能力实现新跨越。认真落实金融服务实体经济、防控金融风险、深化金融改革三项任务，促进经济和金融良性循环。加强信用体系建设，优化金融环境，推进银行、证券、基金、保险、担保等金融业态的合作与发展，打造多层次资本市场体系和金融要素相对完善的宏观金融战略平台。加强金融队伍建设，提高领导干部、企业负责人金融知识和政策水平。正确认识债务、理性分析债务，理智融资、适度举债、良性互动，形成安全、协调的融资、投资、收益及还本付息的理性计划。建立重大项目信息共享机制，促进重大项目与金融机构有效对接，不断满足重大项目融资需求。

　　（六）坚持人与自然和谐共生，确保美丽临沧建设实现新跨越。树牢绿水青山就是金山银山理念，加快构建生态功能保障基线、环境质量安全底线、自然资源利用上线“三大红线”，打好污染防治攻坚战，主动当好生态文明建设排头兵。建立健全以自然保护区、国家公园、森林公园为重点的生态保护体系，保护好森林生态系统、湿地生态系统和珍稀濒危物种资源，构建生态廊道和生物多样性保护网络。实施退耕还林、陡坡耕地生态治理、河道治理和农村能源建设等重要生态工程。到2020年，陡坡耕地退耕还林还草应退尽退。到2022年，全市森林覆盖率达70%左右。抓好大气、水、土壤污染防治，严格控制使用化肥农药，减少农业面源污染。抓好南汀河流域、澜沧江沿线、边境一线生态产业化建设。

　　（七）着力提升文化软实力，确保文化繁荣发展实现新跨越。加快构建现代公共文化服务体系，深入实施文化惠民工程、广场文化品牌工程，着力打造具有临沧特点、国家水准、国际影响的文化品牌，加快建成世界佤文化中心。到2022年，现代公共文化服务体系基本建成，艺术创作更加繁荣，文化遗产和非物质文化遗产得到有效保护和传承，文化市场体系更加完善，文化产业实现长足发展。发展竞技体育事业，推动全民健身、全民健康深度融合。

　　（八）加快推进幸福临沧建设，确保民生保障事业实现新跨越。补齐民生短板，全面建成覆盖全民、统筹城乡、可持续、多层次的社会保障体系，提高基本公共服务水平，让各族群众有更多获得感、幸福感、安全感。实现更高质量、更加充分的就业，构建和谐的劳动关系。建立一批院士、教授、专家工作站，引进各行各业人才。坚持教育优先，推进各类教育协调发展，不断提高教育教学质量，办好人民满意教育。推进健康临沧建设，深化医药卫生体制改革，不断改善医疗卫生服务设施。坚持中西医并重，发展中医药和大健康产业。加强重大疾病防控和卫生应急体系建设，积极创建国家卫生城市。实施好妇女、儿童发展纲要。支持社会养老事业发展。开展质量提升行动，实施食品药品安全战略，加快安全生产领域改革发展。认真落实党的民族宗教政策，继续实施改善沿边群众生产生活条件三年行动计划，加快建设民族团结进步示范区。加大水库移民后期扶持力度。牢固树立总体国家安全观，加强和创新社会治理，不断改进信访工作，有效预防和化解社会矛盾。强化社会治安防控体系建设，依法打击和惩治黄赌毒黑拐骗等违法犯罪活动。加强双拥工作，充分发挥“五位一体”边境管控机制作用，切实维护边境稳定。

　　（九）增强政府公信力和执行力，确保政府自身建设实现新跨越。坚持党的全面领导，坚定不移维护党中央权威和集中统一领导，提高政治站位和执政本领。主动接受市人大及其常委会的法律监督、工作监督和市政协的民主监督，自觉接受审计、监察监督和民主党派、人民群众、社会舆论监督。建设法治政府，严格按照法定权限和程序履行职责。深化政府机构和行政体制改革，转变政府职能，进一步简政放权，创新监管方式，加强政府诚信建设，全面推进政务公开，推行“互联网+政务服务”，推动政府治理、社会调节、居民自治良性互动，建设廉洁政府。持续反“四风”，全力打造最优质的“五个环境”，建设人民满意的服务型政府。

　　三、2018年重点工作

　　2018年，我们将全面落实党的十九大精神，以习近平新时代中国特色社会主义思想为指导，进一步落实习近平总书记对云南发展的重要指示精神，贯彻落实中央经济工作会议、中央农村工作会议、全国扶贫开发工作会议和省委十届四次全会、省十三届人大一次会议、市委四届三次全会精神，坚持稳中求进工作总基调，坚持新发展理念，按照高质量发展要求，统筹推进“五位一体”总体布局，协调推进“四个全面”战略布局，坚持以供给侧结构性改革为主线，大力推进改革开放，推动质量变革、效率变革、动力变革，在打好防范化解重大风险、精准脱贫、污染防治攻坚战方面取得扎实进展。加强和改善民生，促进经济社会持续健康发展。

　　全市经济社会发展主要目标建议为：地区生产总值增长10%；固定资产投资增长20%；规模以上工业增加值增长13%；地方一般公共预算收入增长10%；地方一般公共预算支出增长10%；社会消费品零售总额增长12.5%；外贸进出口总额增长15%；城镇常住居民人均可支配收入增长9%；农村常住居民人均可支配收入增长10%；招商引资市外到位资金增长12%。

　　围绕上述目标，重点抓好以下工作：

　　（一）狠抓固定资产投资，加快“五网”基础设施建设

　　继续实施“四个一百”重点项目建设，注重项目前期，做优项目储备，确保新开工一批、竣工一批。

　　强化综合交通建设。围绕“县县通高”目标，全面推进玉溪至临沧、临翔至清水河等10条（段）高速公路建设，争取开工云县至南涧高速公路，完成高速公路投资160亿元以上。推进“四好农村公路”建设，实施建制村窄路加宽，完成直过民族、沿边自然村道路硬化工程2000公里以上。确保大理至临沧铁路完成投资15亿元以上，争取开工临沧至清水河铁路，扎实推进临沧至普洱、临沧至芒市铁路项目前期工作。加快凤庆、永德机场建设和孟定机场项目前期工作。

　　加强水利、能源和通信建设。新开工10件中型和小（一）型水库、2件水系连通、2件澜沧江水资源综合开发利用和1件中型灌区工程；加快34件续建水利项目建设，确保竣工验收5件重点水源工程；推进21件重点项目前期工作；建设山区“五小水利”工程1.7万件；完成16万人农村饮水巩固提升任务。完成规模以上水利水电固定资产投资120亿元。新增高效节水灌溉面积5万亩以上，力争有效灌溉保障率达52%。加快推进中缅电网互联互通项目前期工作。开工天然气管道（临沧）支线项目。实施宽带中国“城市千兆到户”“农村百兆到户”工程。加快建制村直接通邮，推进县乡村三级农村物流网络建设。

　　（二）狠抓精准扶贫精准脱贫，加快脱贫攻坚任务落实

　　着力加强作风建设，确保3个县（区）、4.8万贫困人口脱贫摘帽。抓紧2016年扶贫搬迁扫尾工作，确保3月底前全面完成2017年52个易地扶贫搬迁安置点建设，6月底前实现100%入住。实施好5.2万户农村危房改造工作。优先安排农村道路、人畜饮水安全等项目，补齐基础设施短板。继续扶持贫困人口人均有5亩以上产业基地。开展农村劳动力转移就业培训，新增转移就业15万人次。深化农村集体土地改革，增加贫困人口的财产性收入。实施退耕还林还草工程，带动农村人口稳定增收。落实好“健康扶贫”30条措施，让困难群众“看得起病、方便看病、看得好病、尽量少病、病不致贫”。实现适龄少年儿童无辍学，落实好资助政策、免费职业教育招生托底政策。对“直过民族”和人口较少民族开展普通话普及，实现农业生产技术和职业技能培训全覆盖。创建全国民族团结进步示范市，扎实推进第二轮改善沿边群众生产生活条件三年行动计划。

　　围绕“两不愁、三保障”，加强建档立卡贫困户动态管理和成果运用，分类推进精准扶贫措施到村到户到人。注重扶志与扶智并举，挖掘脱贫潜能。关心扶贫干部，加强队伍培训和管理，提高攻坚能力。强化资金管理，整合涉农资金。强化跟踪问效和审计监督，开展扶贫领域专项治理。

　　（三）狠抓产业培育建设，加快做大经济总量

　　推进工业强市战略，把发展经济的着力点放在实体经济上，实施100个规模以上工业固定资产投资项目，实现非电工业投资增长10%。完成全社会用电量32.5亿千瓦时。推动工业企业向园区聚集发展、集群发展，确保工业园区规模以上工业增加值增长15%以上。帮扶民企解困，推动实体企业释放产能、降低成本、提高效益。指导企业参与电力市场化交易6亿千瓦时以上，降低企业用电成本。扶强做大国企，支持企业上市，力争新增规模以上工业企业15户以上。企业年报公示率保持在90%以上。

　　积极发展绿色循环经济，强化环保目标责任落实。引导企业开展清洁生产和资源综合利用，创建节水标杆企业和资源循环化利用园区。

　　推进电子商务、物流等现代服务业发展。加快沧源国际旅游度假区建设，启动翁丁景区、崖画谷4A级景区创建，推进13个民族特色旅游村、15个营地建设，启动建设3个美食街区，建设5条旅游公路、30个观景台、8个游客服务中心和一批旅游厕所。巩固提升“国家森林城市”和“全国森林旅游示范市”创建成果，推进森林城市与旅游融合发展。抓好边境旅游示范区建设，融入全省“一部手机游云南”数据平台和“智慧旅游”建设，实现旅游业总收入231亿元，增长40%。

　　（四）狠抓沿边开发开放，加快促进对缅开放合作

　　启动孟定清水河口岸经济区双创孵化园及综合保税标准厂房建设项目、亚行贷款2.5亿美元综合发展项目。推进临沧边合区国际产能合作示范区建设，完善边境口岸物流设施。

　　推动清水河口岸迁建和对等开放工作，争取缅甸清水河口岸联检设施纳入对外援建计划。做好南伞、永和口岸升格开放层级的争取工作，加快南伞口岸货运通道联检设施和永和口岸服务中心建设。加快沿边金融综合改革试验区建设，加强跨境金融开放合作和业务创新。

　　坚持“四个先行”，推动滚弄大桥援建项目开工建设，整合商务、外事、侨务等资源，充分发挥驻缅商务代表处作用，鼓励国内企业走出去，引进缅方企业入驻临沧。加强与缅甸经贸、劳务、技术、文化、医疗、教育交流合作，推进替代种植向替代产业转型发展。

　　突出精准招商，瞄准世界500强、国内500强和行业龙头企业，重点推出100个高原特色产业、100个“五网”基础设施建设和100个沿边对外开放招商项目。

　　（五）狠抓城镇规划建设，加快提升城镇生态宜居水平

　　完成市域城镇体系规划、临沧市城市总体规划、临沧中心城区东部拓展规划、物流枢纽规划，加快多规合一大数据平台建设。完成历史文化街区划定和历史建筑确定，完善城市配套功能。

　　推进临沧中心城区东拓发展，加快建设临沧民族风情美食文化大观园、亚微会展中心。全力抓好保障性住房扫尾入住工作。新开工棚户区改造1.69万套（户）。扩大住房公积金制度覆盖面，新增缴存人员1.2万人以上。实现商品房销售面积增长10%。推进地下综合管廊、海绵城市建设。抓好6个特色小镇建设。完成城镇建设投资350亿元。

　　实施园林城市、森林城市、文明城市、卫生城市、智慧城市、平安城市“六城同创”。抓好国家土地例行督察问题整改工作。继续实施城乡“四治三改一拆一增”人居环境提升行动。完善城市基础设施，全部打通城市现存“断头路、丁字路”，新建一批城市公厕、停车场，城区公厕数量达到每平方公里5座以上。加快推进城市公交改革。积极抓好临沧中心城市国家级园林城市申报工作，建设一批城市森林公园，推进城市公园和道路绿化管理市场化、专业化改革。

　　（六）狠抓农业农村发展，加快实施乡村振兴战略

　　推进农村产业融合，着力深化农业供给侧结构性改革，坚持用工业化理念谋划农业，塑造“绿色牌”，推动农业“绿色革命”。实现粮食总产量108万吨。巩固提升核桃、坚果、烤烟、咖啡、橡胶、畜牧等传统产业，积极发展蚕桑、中药材等新兴特色产业，完成烤烟收购75万担。做大“茶、果、糖、菜”等传统主导产业，打造“一村一品、一县一业”产业发展新格局，大力发展特色产品精深加工，新培育20户以上农业产业龙头企业、50户以上休闲农业经营主体。加快推进云南坚果交易中心建设，办好第八届国际临沧澳洲坚果大会，力争常设机构设立临沧。积极发展“互联网+现代农业”，推进农业物联网试点工程建设，尽快实现临沧农产品“下山、进城、出海、上网”。

　　深入实施“七改三清”人居环境提升行动，抓好传统村落保护与建设，打造一批独具临沧特色的“鲜花盛开的村庄”。加强农村思想、道德、公共文化建设，培育文明乡风、良好家风、淳朴民风。创新乡村治理体系，走乡村善治之路。培养造就一支懂农业、爱农村、爱农民的“三农”工作队伍。

　　严格环保执法，推进地质灾害防治体系建设，不断加强公益林、天然林、自然保护区等重点保护工作，统筹抓好山水林田湖草系统治理。完成新一轮退耕还林还草40.3万亩，新造林60万亩，陡坡地生态治理1万亩。创建森林村庄100个，建成示范精品森林村庄16个。

　　（七）狠抓财税金融工作，加快提升资金保障能力

　　建好融资平台。以市国资公司运营的财务结算中心（资金池）为核心平台，逐步覆盖市级国有企业，健全其预算、归集、备付金等运行机制，编制好年度、月度融资计划、还款计划，以“分贷统还”“分贷分还”“统贷统还”等形式开展融资，形成现金流的良性循环机制，确保任何时候资金池都有必要规模的安全保障资金。

　　推动融资平台市场化转型，发挥银行融资主渠道作用，确保新增银行贷款80亿元以上。加快推动市工投公司等3户AA级主体信用评级工作，全力推进各平台公司创新融资。

　　加强财源培植，强化税收征管，提高财政收入质量，全市非税收入占地方一般公共预算收入比重下降5个百分点。积极争取上级专项资金、专项建设基金、专项债券等支持。

　　规范举债行为，强化预算约束，加强预算管理，统筹可用财力，加强信用体系建设，积极探索还本付息预拨机制，着力构建良好的还本付息机制。加强金融专业人才队伍建设。不断提高债务管理水平，确保不发生金融风险。

　　（八）狠抓保障和改善民生，不断提高人民群众获得感

　　深化教育改革。办好学前教育，实施“一村一幼”建设。抓好义务教育，实施“全面改薄”等工程建设，完成沧源自治县、镇康县国家义务教育基本均衡检查验收，确保义务教育巩固率达94%。加快高中教育普及工作，提高职业教育、高等教育办学质量，抓好民族教育、特殊教育和继续教育。启动临沧职业学院建设。积极支持和规范民办教育。

　　加大力度解决再就业、创业问题，落实贷免扶补政策，确保城镇新增就业人数2.5万人。进一步完善城乡保险制度，抓好保险扩面工作，实施“同舟计划”，新开工项目100%参加工伤保险。

　　完成市人民医院三级甲等医院创建工作，加快市中医医院（市佤医医院）等一批项目建设，实施县级中心医院提质达标晋级行动，全力提升城乡医疗服务能力。加快推进紧密型县乡村医疗卫生服务一体化管理、医保支付方式改革及异地就医即时结算。深化医药卫生体制改革，推进分级诊疗、现代医院管理、全民医保、药品供应保障、综合监管等制度建设，控制医疗费用不合理增长。

　　加强文化建设，完善市、县、乡、村文化设施网络。强化全民阅读、农村书屋等文化惠民工程建设。推进优秀传统文化传承发展工程，实施“国门文化形象”建设工程。办好第六届亚微节、美食文化周、博尚菜花文化周等文化旅游推介活动。大力发展体育事业，办好第十五届省运会、第十一届省残运会、第十一届省民运会等重大赛事活动。

　　推进科技创新，抓好“科技入滇”，形成“科技入临”并实现成果转化。完成申报创建国家可持续发展议程创新示范区工作。开展好第四次全国经济普查和第三次全国土地调查工作。

　　大力推进法治临沧建设，抓好“七五”普法工作，构建立体化的社会治安防控体系，打好禁毒防艾人民战争，建设“平安临沧”。加强安全生产专项整治，创新食品药品监管机制。落实电站库区移民安置扫尾和后期扶持政策。抓好群众来信来访工作，强化社会矛盾纠纷排查化解，防范群体性事件发生。着力提升防灾减灾能力。进一步加强双拥工作，推进军民融合发展，加快“智慧边境”建设，抓好抵边人居环境提升，维护边境稳定和谐。

　　落实好社会救助、社会福利、慈善事业、优抚安置等制度，关爱农村留守儿童和妇女、老年人，进一步做好老龄、保密、气象、水文、地震、地方志、参事、档案、供销、外事、侨务和对台工作，支持工商联、工会、共青团、妇联、残联、红十字会、文联、社科联、科协等人民团体的工作，确保各项社会事业取得新进步。

　　继续办好10件惠民实事。

　　（九）狠抓政府自身建设，加快建设服务型政府

　　持续深化“两学一做”学习教育、“党的光辉照边疆，边疆人民心向党”实践活动和知恩感恩教育，扎实开展“不忘初心、牢记使命”主题教育。以“六不思维”直面矛盾，集中整治“六种顽疾”，教育整改“六不之人”，持续整治“三难”，巩固提升“三好”。深入纠正“四风”，构建“亲清”政商关系。大力弘扬“临沧作风”，着力建设“沧江铁军”。

　　继续深化“放管服”改革，推进商事制度改革，有序推进事业单位分类改革，不断完善市场准入负面清单制度。加快推进“互联网+政务服务”，构建审批事项最少、审批时间最短、监管依法依规、服务热情周到、“一颗印章管审批”、到政府办事最多跑一次的临沧政务软环境。

　　抓好省委巡视反馈意见整改落实。执行好政府工作季度审计、领导干部自然资源资产离任审计、政府重要工作法制办审查、第三方法律审查制度，严格按照法定权限、法定程序行使职权，落实行政执法责任追究制度，加强诚信体系建设，提升政府公信力。

　　加强行政权力运行制约和监督，强化政务公开。自觉接受市人大及其常委会的法律监督、工作监督和市政协的民主监督，做好人大代表建议和政协提案办理工作。自觉接受审计、监察监督和民主党派、人民群众、社会舆论监督。

　　各位代表、同志们，回顾过去，我们克难攻坚，硕果累累；迈入新时代，展望新蓝图，咱们临沧斗志昂扬，信心百倍。让我们紧密团结在以习近平同志为核心的党中央周围，以习近平新时代中国特色社会主义思想为指引，在省委、省政府和市委的坚强领导下，凝心聚力，开拓创新，跨越发展，为夺取全面建成小康社会的伟大胜利而努力奋斗！
